# A novel reassortant strain of the infectious bursal disease virus (IBDV ASPVB) from Iraqi broiler farms: A first-time molecular and histopathological investigation revealing new insights

**DOI:** 10.5455/javar.2025.l906

**Published:** 2025-04-30

**Authors:** Ammar Dhari Abdel Fattah, Samer Sadeq Hameed

**Affiliations:** 1 College of Veterinary Medicine, University of Baghdad, Baghdad, Iraq

**Keywords:** Broiler farms, immunosuppressive, phylogenetic analyses, reassortant strain

## Abstract

**Objective:**

The objective of this investigation was to identify and detect the reassortant infectious bursal disease virus (IBDV) strain from broiler farms suspected of being infected.

**Materials and Methods:**

The broiler yielded 72 samples, including the spleen and bursa of Fabricius. The tissues underwent histological examination before being used in a typical PCR molecular investigation.

**Results:**

The strain was subsequently termed IBDV ASPVB. The IBDV ASPVB strain in Iraq has been identified as a novel reassortant strain based on the results of PCR, sequencing, and phylogenetic analysis of partial segments A and B. Segment A of this strain is derived from the highly pathogenic IBDV strain. In contrast, segment B is derived from other field reassortant strains. Infection with this strain might result in minor clinical symptoms but substantial damage to lymphoid organs, leading to compromised immunological responses.

**Conclusion:**

As a result of ongoing evolution, this study demonstrates that IBDV in Iraq exhibits a wide range of histological, genetic, and phenotypic variation; to our knowledge, this paper represents the first report of reassortant IBDV in Iraq.

## Introduction

Infectious bursal disease (IBD), often called Gumboro disease, is a highly contagious infectious ailment that typically affects young chickens between the ages of 3 and 6 weeks after maternal antibodies decline. However, birds aged 0–3 weeks frequently exhibit a milder or subclinical form of the disease [1]. The virus’s impact on the bursa of Fabricius can lead to additional complications. The infectious bursal disease virus (IBDV) leads to the reduction of lymphoid cells in the bursa. If the reduction happens within the first 2 weeks of life, it might cause a considerable decrease in the production of antibodies [2].

Infectious bursal disease virus is a type of virus that contains double-stranded RNA. The *Avibirnavirus* genus and the Birnaviridae family classify it. There are two different serotypes of this virus. Serotype 1 induces pathology in hens, but type 2 is inherently non-pathogenic. Serotype 1 consists of both classical and variant antigenic subtypes. We can further categorize the classical subtypes into three pathotypes: mild/attenuated, virulent, and very virulent IBDV [3,4]. The virus has a genome divided into two segments: A and B. An icosahedral-shaped capsid, not an envelope, encloses it. This virus is responsible for causing an illness in young chickens that weakens their immune system. In nature, it is usual for viruses with segmented genomes to undergo genetic material exchange when they simultaneously infect a single cell [5]. Segment A consists of two partially overlapping open reading frames (ORFs). The smaller ORF encodes the VP5 protein, while the larger ORF encodes the polyproteins VP2-VP4-VP3. These polyproteins are then processed into VP2, VP4, and VP3 through autolytic cleavage [6,7]. VP2 functions as a significant structural protein and a host-protective immunogen that is strongly linked to IBDV cell tropism, antigenic variation, and virulence. Segment B exclusively encodes the VP1 protein, which exhibits RNA-dependent RNA polymerase activity. This activity is crucial for viral genome replication, translation, and virulence [8,9].

Recombinant IBDV strains containing segment A from a highly virulent IBDV and segment B from either a serotype 1 or serotype 2 virus have been documented [10,11]. They exhibit lower pathogenicity in comparison to conventional vvIBDV. Phylogenetic investigations have indicated that intersegment homologous recombination events across distinct strains of IBDV may take place [12,13]. This mechanism enables viruses to acquire novel harmful traits and antigenic combinations, hence enhancing their evasion of the immune system and transmission between other species [14]. IBDV reassortants originating from Asia, North America, Africa, and Europe have been previously documented [15–20]. This study aims to identify and detect the reassortant infectious IBDV strain from broiler farms that may be infected using histology and molecular methods.

## Materials and Methods

### Ethical approval

The investigation was carried out according to the animal welfare code in Iraq (577 on 12/3/2023).

### Filed sample collection

A total of 72 samples were collected from the suspected infected broiler, encompassing various tissues, including the bursa of Fabricius and the spleen. These samples were gathered from 20 poultry farms located in different provinces in Iraq, namely Baghdad, Al Kut, Al Anbar, Al Najaf, Dialya, and Kirkuk. The collection focused on chickens exhibiting IBD symptoms such as vent picking, depression, trembling, white watery diarrhea, and ruffled feathers. The collected samples were carefully dissected under sterile conditions, and each was appropriately labeled. To preserve their integrity, the samples were promptly transported on ice to the laboratory and then stored at –20°C until further processing.

### Histopathological examination

The histological sections were prepared in the Department of Pathology and Poultry Illnesses in the College of Veterinary Medicine. The histological sections were produced as follows: The specimens were preserved by immersing them in a 10% neutral buffered formalin solution for 24–48 h. After washing and dehydration, the residual formalin was eliminated, and the samples were subsequently immersed in a solution of graded ethanol with a concentration of 90%. The ethanol solution was removed through 3 rounds of clearing. The infiltration process was carried out twice at a temperature of 56°C–58°C. The embedding method was carried out by placing the samples in containers filled with liquid paraffin at a temperature range of 56°C–58°C. The samples were then left at room temperature until they became solid. Subsequently, the samples were subjected to freezing and then cut into sections with a thickness of 5 μm using a rotary microtome. Ultimately, the samples were immersed in a water bath set at 50°C–55°C for staining.

### Extraction of total RNA from tissue

The AccuZolTM kit from BIONEER was utilized to extract total RNA from tissue samples of the bursa of Fabricius. AccuZol was applied to each tissue sample, enhancing the cell lysis process. Subsequently, chloroform was introduced into the mixture, which was vigorously agitated before being subjected to centrifugation. Subsequently, the RNA obtained was subjected to drying, centrifugation, and precipitation using 80% ethanol. Afterward, the RNA pellet was dissolved in RNase-free water or a suitable buffer and then incubated for 10 min at 55°C–60°C. The extracted RNA samples were stored at a temperature of –20°C to ensure long-term stability.

### First-strand cDNA synthesis protocol

The retro-transcription phase was conducted using the EasyScript™ kit (Abm, Canada) in accordance with the manufacturer’s instructions. First, the RNA samples and all reagents should be thawed on ice with thorough mixing for each solution. The following recipe of the reaction mixture is shown in Table 1. Providing that all handling steps were performed on ice to avoid loss in enzyme activity and maintain the stability of the RNA samples.

**Table 1.** Ingredients of the reaction mixture that was utilized to create the initial strand of cDNA.

Component	Volume	Final concentration
Total RNA or poly(A) + mRNA	Variable	1.0 ng–2.0 μg/rxn 1.0 pg–2.0 ng/rxn
Oligo (dT) (10 μM) or random primers (10 μM) orgene-specific primer	1.0 μl	
	1.0 μl	
	Variable	
dNTPs (10 mM each)	1.0 μl	500 μM
5X RT Buffer	4.0 μl	1X
RNasin (40 U/μl)	0.5 μl	20 U/rxn
EasyScriptTM	1.0 μl	200 U/rxn
Nuclease-free	H_2_O Up to 20 μl	------

rxn: reaction mixture, U: units.

Following the completion of the cDNA first-strand synthesis reaction, it is necessary to thoroughly mix the components in the tube and then collect them using pulse centrifugation for a duration of 30 sec. The reaction mixture was subjected to incubation at a temperature of 25°C for a duration of 10 min when using random primers. However, this incubation step can be skipped if Oligo (dT) or gene-specific primer is utilized. The cDNA synthesis procedure was performed by allowing the reaction mixture to incubate for 50 min at a temperature of 42°C. Ultimately, the reaction was terminated by subjecting it to a temperature of 85°C for 5 min and thereafter cooling it on ice. The recently produced initial-strand cDNA is prepared for immediate use in subsequent procedures or for extended preservation at a temperature of −20°C.

### Primer design

Two sets of primers were designed to amplify segments A and B [21]. The synthesis of each of the designed primers was performed by DNA Integrated Biotechnology (IDT Co., Canada). The primer for segment A is presented in Table 2, while the primer for segment B appears in Table 3.

**Table 2.** Primer sequences used for the amplification of a partial sequence of segment A.

Primer	Sequence (5’→ 3’)	Size of amplicon (bp)
Forward primer	CTTCCAAGGGAGCCTGAGTG	993 bp
Reverse primer	ACCACCGGTACAGCTATCCT	
Forward primer	GAGCCTAGCAGTGACGATCC	739 bp
Reverse primer	GCTGTTCAGTGCTTTGGGTG	
Forward primer	CACCCAAAGCACTGAACAGC	917 bp
Reverse primer	AGCTACCCATTCCGGTGTTG	

**Table 3.** Primer sequences used for the amplification of a partial sequence of segment B.

Primers	Sequence (5’→ 3’)	Size of amplicon (bp)
Forward primer	AAGCAAGATCTCAGCAGCGT	632
Reverse primer	AAGGCTTGTCATCCTCACCG	
Forward primer	TTGTGGCCATGAAGGAGGTC	286
Reverse primer	ATTGTCTCTCCCTTGGTGCG	
Forward primer	CGCACCAAGGGAGAGACAAT	917
Reverse primer	ATGTAGCTGACCACCCAAGC	
Forward primer	GTACCTGAGTGGGGGTGTTG	546
Reverse primer	CCACTCAGTCCGGCTTCATT	
Forward primer	AATGAAGCCGGACTGAGTGG	382
Reverse primer	CCATTGGTCTGCTCGTTCCT	

## Results

### The clinical signs, gross lesions, and histopathological examination

**Figure 1.**
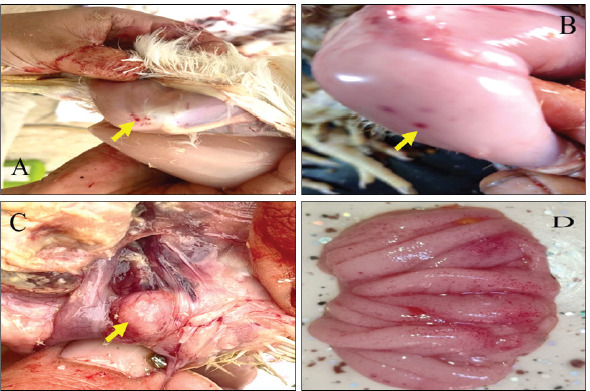
A and B: Chicken infected with IBD exhibits that hemorrhages are evident in the thigh muscle (blue arrows). C: Chicken infected with IBD exhibits enlargement and hemorrhage in the bursa of Fabricius. D: Enlarged, edematous, and hemorrhagic the bursa of Fabricius.

**Figure 2.**
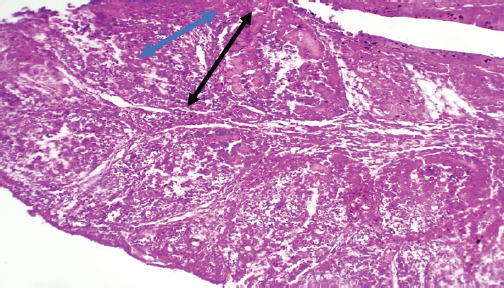
Section of bursa showed follicular necrosis (black arrow) accompanied with perifollicular cellular infiltration (blue arrow) composed mainly of lymphocytes and heterophils (H&E stain X100).

**Figure 3.**
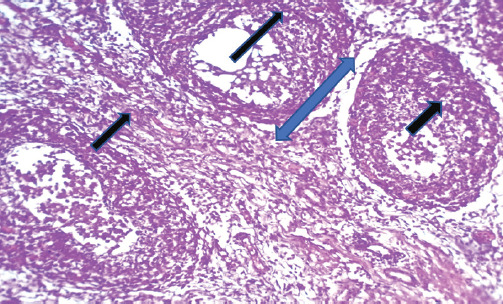
Section of bursa showed lymphoid depletion with evidence of medullar necrotic debris (black arrow) together with numerous capillaries and mild MNCs infiltration of the inter follicular tissue (blue arrow) (H&E stain X100).

**Figure 4.**
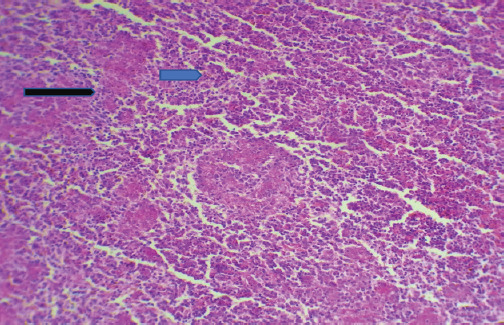
Section of spleen showed extensive necrosis of splenic white pulp mainly in periarteriolar sheath) blue arrow) with mild congestion in the red pulp (black arrow) (H&E stain X100).

**Figure 5.**
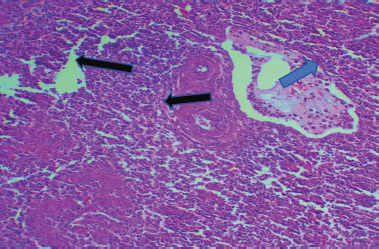
Section of spleen showed multifocal necrotic areas) blue arrow) with sinus congestion and dilation (black arrow) (H&E stain X100).

The chicken displayed clinical manifestations of IBD, including the presence of white watery diarrhea, ruffled feathers, vent plucking, loss of appetite, dehydration, and heightened water intake. In addition, there were observable hemorrhages present on the thigh muscle (Fig. 1A and 1B). The bursa of Fabricius exhibited a twofold increase in size, accompanied by the presence of a yellowish gelatinous substance that may envelop it. Additionally, there may be instances of hemorrhaging observed on its surface (Fig. 1C and 1D). The results of histopathological examination of the bursa revealed mild follicular necrosis accompanied by perifollicular cellular infiltration of lymphocytes and heterophils (Fig. 2). Severe lesion showed lymphoid depletion with evidence of medullary necrotic debris together with numerous capillaries and mild MNCs infiltration in the interfollicular tissue (Fig. 3). There is modest congestion in the red pulp and widespread necrosis of the splenic white pulp, primarily in the periarteriolar sheath (Fig. 4). Another section of the spleen showed multifocal necrotic areas with sinus congestion and dilation (Fig. 5).

### The results of the molecular analysis with conventional PCR

**Figure 6.**
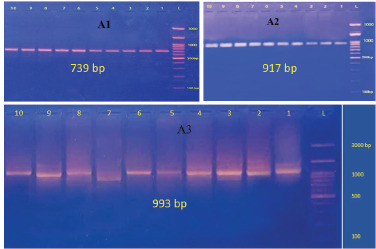
Molecular detection of infectious bursal disease virus (segment A). The amplified findings were visualized using ethidium bromide staining. Perform agarose gel electrophoresis to analyze the PCR results. The DNA ladder is composed of DNA fragments that are 3,000 bp in length. Lanes 1–10 have positive samples with an amplicon length of 739 bp for segments (A) (A1). Lanes 1–10 also contain positive samples with an amplicon length of 917 bp for segments (A) (A2). Finally, Lanes 1–10 contain positive samples with an amplicon length of 993 bp for segments (A) (A3).

**Figure 7.**
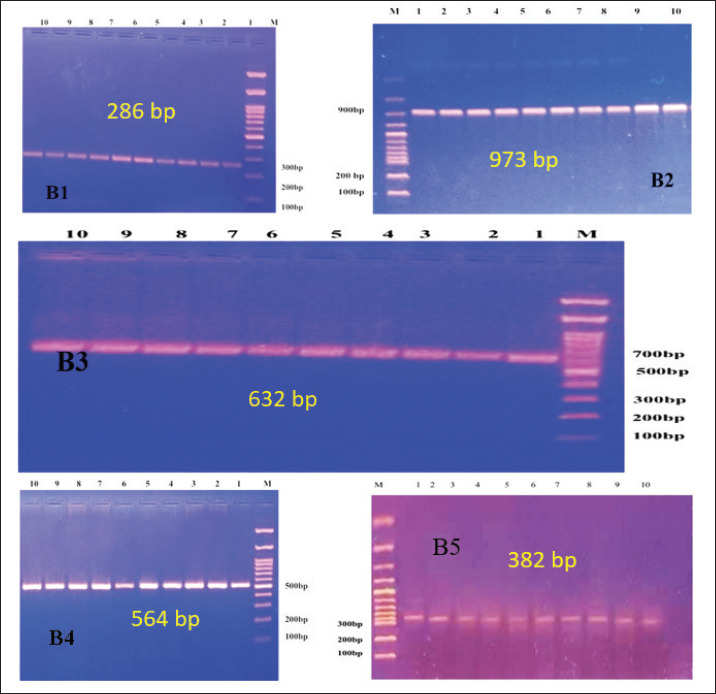
Molecular detection of infectious bursal disease virus (segment B). The amplification results were then stained with ethidium bromide. Perform agar gel electrophoresis of PCR products. A 1500 bp DNA ladder is present. The samples in lanes 1 to 10 show positive results for different segments of size 286 bp (B1), 973 bp (B2), 632 bp (B3), 564 bp (B4), and 382 bp (B5).

PCR was used in this section of the investigation to confirm the presence of IBDV in samples of bursa of Fabricius tissue that had been IBD; 2649 bp constituted the PCR product for the partial segment A, which contains the VP2 variable region (Fig. 6). For partial section B, the PCR result was 2763 bp (Fig. 7).

### Phylogenetic sequence analysis

**Figure 8.**
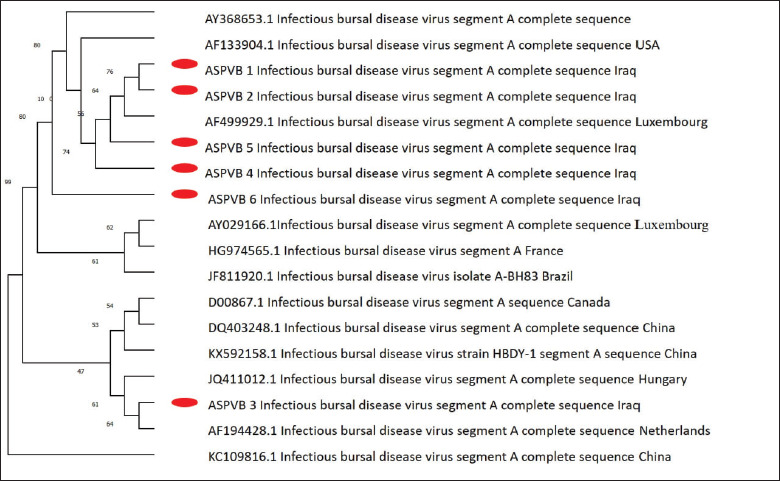
Phylogenetic analysis of segment A nucleotides from this study’s IBDV isolates and NCBI strains. MEGA 5’s neighbor-joining approach built the tree. Bootstrapping values from 1,000 replications are above the branches. Red dots represent this study’s IBDV strains.

**Figure 9.**
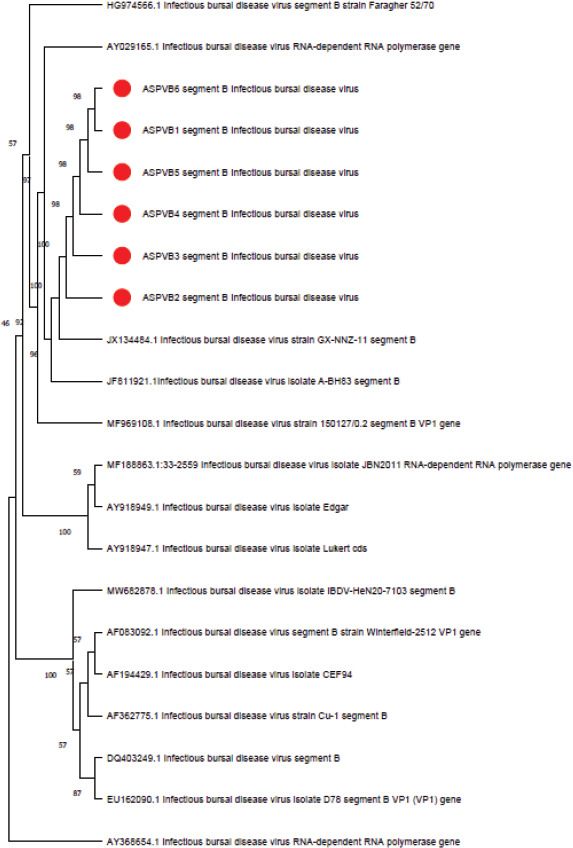
Examining the sequence of nucleotide for segment B from IBDV isolates found in this investigation and IBDV NCBI strains through analysis of phylogeny. The tree was built utilizing the technique known as neighbor-joining in MEGA 5 software. The numbers displayed over each branch represent bootstrap values derived from 1,000 replications. The red dots represent the IBDV strains that were discovered in the present investigation.

The sequences of segments A and B of the positive isolate that were submitted to NCBI revealed similarities to isolates in the bank gene and subsequently assigned accession numbers (Tables 4 and 5). The phylogenetic analysis confirmed that the two segment sequences have different origins, where segment A revealed that the 6 IBVD Iraqi isolates are closely related to very virulent strains, and for segment B the Iraqi isolates are closely related to reasserting and/or attenuated vicinal strains (Figs. 8 and 9; Tables 6 and 7).

**Table 4.** Displays the sequences that were submitted to NCBI and subsequently assigned accession numbers.

Isolate	Accession number	Segment
A1	PP763407.1	A
A2	PP763408.1	A
A3	PP763409.1	A
A4	PP7634010.1	A
A5	PP7634011.1	A
A6	PP7634012.1	A

**Table 5.** Displays the sequences that were submitted to NCBI and subsequently assigned accession numbers.

Isolate	Accession number	Segment
B1	PP795431.1	B
B2	PP795432.1	B
B3	PP795433.1	B
B4	PP795434.1	B
B5	PP795435.1	B
B6	PP795436.1	B

**Table 6.** Genbank sequences that have the highest percent identity with the isolates of the study segment A.

No.	Accession number	Year	Country	Pathotype	Isolate	Percentage of identity
1.	AF499929	2004	Nigeria	Very	A1	92.8
				virulent	A2	92.1
				(vv)	A3	91.8
				variant	A4	91.7
					A5	91.7
					A6	91.7
2.	JF811920	2005	Brazil	Very	A1	92.9
				virulent	A2	92.6
				IBDV	A3	91.6
					A4	91.7
					A5	91.7
					A6	93.7
3.	HG974565	2015	France	Very	A1	92.7
				virulent	A2	92.4
				IBDV	A3	91.7
					A4	91.4
					A5	91.4
					A6	91.9
4.	AF133904	2000	USA	Variant	A1	92.6
				strain	A2	91.9
					A3	91.6
					A4	91.4
					A5	91.8
					A6	91.7
5.	KX592158	2017	China	Very	A1	92.9
				virulent	A2	92.6
				IBDV	A3	92.0
					A4	91.8
					A5	91.8
					A6	92.1

**Table 7.** Genbank sequences that have the highest percent identity with the isolates of the study segment B.

No.	Accession number	Year	Country	Pathotype	Isolate	Percentage of identity
1.	MF969108	2018	Algeria	Reassortant	B1	95.6
					B 2	95.5
					B3	95.3
					B4	95.6
					B5	95.4
					B6	95.5
2.	MF188863	2017	South	Reassortant	B1	94.9
					B2	94.9
					B3	94.7
					B4	95.0
					B5	94.8
					B6	94.9
3.	JX134484	2012	China	Reassortant	B1	97.3
					B2	97.2
					B3	97.1
					B4	97.3
					B5	97.1
					B6	97.2
4.	JF811921	2011	Brazil	Vaccine	B1	96.3
				strains	B2	96.0
					B3	95.8
					B4	96.1
					B5	95.9
					B6	95.9
5.	AY029165	2004	USA	Reassortant	B1	95.8
					B2	95.8
					B3	95.6
					B4	95.9
					B5	95.7
					B6	95.4
6.	MW682878	2021	China	Attenuated	B1	95.2
				strain	B2	95.2
					B3	95.0
					B4	95.2
					B5	95.1
					B6	95.2
7.	DQ403249	2007	China	Attenuated	B1	95.1
				strain	B2	95.0
					B3	94.9
					B4	95.1
					B5	95.0
					B6	95.0

### Discussion

IBDV’s virulence is associated with several genes, particularly the VP2 gene, which is responsible for encoding the primary structural protein of the viral capsid [22]. This protein is crucial for the virus’s pathogenicity, virulence, and tropism [23]. In the case of IBDV, the VP1 protein is made from two different parts of the virus’s genetic material: segment A and segment B. Segment A encodes other important viral proteins, including VP2, which plays a significant role in viral pathogenicity. Segment B of the IBDV genome encodes the VP1 protein, which is crucial for the virus’s replication and transcription.

Studying these segments and their encoded proteins provides valuable insights into the virus’s biology and helps develop effective control measures and vaccines [24]. This study aimed to analyze suspected outbreaks of IBD in 20 poultry farms across various provinces in Iraq, including Baghdad, Al Kut, Al Anbar, Al Najaf, Diyala, and Kirkuk. The objectives were to identify the specific strains of IBDV present in these cities, explore the potential presence of reassortant IBD strains, and conduct a comprehensive genome analysis of the Iraqi isolates of IBDV. The findings will contribute to a deeper understanding of the circulating viral strains within the country. The IBDV was detected in the bursa of all samples by PCR. The clinical signs and gross lesions observed in this study are consistent with previous research [25,26]. These findings confirm that clinical signs of IBDV are characterized by white, watery diarrhea, dehydration, and increased water consumption.

In this study, the results of histopathological examination of the bursa and spleen revealed mild to severe lesions (follicular necrosis and lymphoid depletion). The extent of lesions in the BF and spleen serves as an indicator and is correlated with the level of pathogenicity of the virus in chickens that are naturally infected [24], also noted these data, which showed a depletion of lymphoid follicles in this investigation. These changes were consistent with prior reports of lymphoid follicle depletion caused by infection with highly virulent IBDV [27,28]. In this study, a portion of the viral genome segments A and B were sequenced, followed by phylogenetic analysis, which revealed that six positive fields were carried out by PCR using segments A and B. The variations in the nucleotide sequences were shown to be associated with the level of virulence and ability to cause disease in the IBDV strains. The results indicated that the nucleotide sequence of segment A of six positive Iraqi isolates was compared to the published sequence of normal IBDV, revealing a significant similarity to vvIBDV strains [29,30].

However, segment B of these isolates was found to be identical to reassortant isolates. The phylogenetic study of the amino acid sequences corroborated these links, as the latest Iraqi IBDVs showed solid genetic similarity to other very vvIBDVs. Phylogenetic analyses based on segment A revealed that the 6 IBVD Iraqi isolates are closely related to Luxembourg, China, Canada, Netherlands, and Hungary strains, and for segment B, the Iraqi isolate is closely related to the Gx-NNZ-11 strain, the A-BH83 isolate, and the 1501270.2 strain, which are reassortant and/or attenuated vaccinal strains. Moreover, this study demonstrates the spread of the reassortant vvIBDV strain among poultry farms in Iraq. Phylogenetic analysis of segments A and B of the IBDV ASPVB of Iraqi isolates provides valuable insights into the virus’s genetic diversity, evolutionary relationships, and epidemiological patterns. This analysis is essential for developing effective vaccines, tracking the spread of the virus, and understanding its pathogenic mechanisms. Using comprehensive sequencing and advanced computational methods, researchers can enhance our knowledge of IBDV and improve its control and prevention strategies.

## Conclusion

The clinical signs, histopathological, and molecular findings collectively support the diagnosis of IBD in suspected chickens. This study reveals a broad genetic and phenotypic diversity of IBDV in Iraq due to continuous evolution. We present, to our knowledge, the first description of the reassortant IBDV in Iraq. This study showed the ongoing prevalence of reassortant IBD in numerous poultry farms. It emphasizes the need for continued wariness and updated strategies in disease management within the poultry industry.

## List of abbreviations

cDNA, complementary DNA

dNTPs, Deoxynucleotide triphosphates

H and E, hematoxylin and eosin

IBD, Infectious bursal disease

IBDV ASPVB, Infectious bursal disease virus isolate ASPVB

MEGA 5, Molecular evolutionary genetics Analysis version 5

MNCs, Mononuclear cells

NCBI, National center for biotechnology information

PCR, Polymerase chain reaction

RT Buffer, reverse Transcription*10 Buffer

vvIBDVs, very virulent infectious bursal disease virus

μm, micrometers

h, hour

sec, seconds

min, minutes.
